# Effects of irrigation scheduling on the yield and irrigation water productivity of cucumber in coconut coir culture

**DOI:** 10.1038/s41598-024-52972-x

**Published:** 2024-02-05

**Authors:** You-li Li, Si-qi Zhang, Wen-zhong Guo, Wen-gang Zheng, Qian Zhao, Wen-ya Yu, Jian-she Li

**Affiliations:** 1 School of Civil and Hydraulic Engineering, Ningxia University, Yinchuan, 750021 Ningxia China; 2 Equipment Research Center, Beijing Academy of Agriculture and Forestry Sciences, Beijing, 100097 China; 3 Beijing Cuihu Agricultural Technology Co., Ltd, Beijing, 100097 China

**Keywords:** Plant sciences, Plant development, Plant genetics

## Abstract

Optimum irrigation scheduling is important for ensuring high yield and water productivity in substrate-cultivated vegetables and is determined based on information such as substrate water content, meteorological parameters, and crop growth. The aim of this study was to determine a precise irrigation schedule for coconut coir culture in a solar greenhouse by comparing the irrigation, evapotranspiration (ET), substrate water content (VWC), as well as the crop growth indices and yield of cucumber, and irrigation water productivity (IWP) under three irrigation schedules: the soil moisture sensor-based method (T-VWC), the accumulated radiation combined with soil moisture sensor-based method (Rn-VWC), and the crop evapotranspiration estimated method using the hourly PM-ETo equation with an improved calculation of Kc (T-ETc). The results showed that the daily irrigation and evapotranspiration amount were the highest under T-VWC treatment, while the lowest under T-ETc treatment. In different meteorological environments, the change in irrigation amount was more consistent with the ET,and the VWC was relatively stable in T-ETc treatment compared with that under T-VWC or Rn-VWC treatments. The plant height, leaves number, leaf area, and stem diameter of T-VWC and Rn-VWC treatments were higher than those of the T-ETc treatments, but there was no significant difference in cucumber yield. Compared with the T-VWC treatment, total irrigation amount under Rn-VWC and T-ETc treatments significantly decreased by 25.75% and 34.04%, respectively ($\hbox {P}<0.05$). The highest IWP values of 25.07 kg m$^{-3}$ was achieved from T-ETc treatment with significantly increasing by 44.33% compared to the T-VWC treatment (17.37 kg m$^{-3}$). In summary, the T-ETc treatment allowed more reasonable irrigation management and was appropriate for growing cucumber in coconut coir culture.

## Introduction

Substrate cultures are widely used for protected cultivation because of their multiple advantages over soil cultivation^1–4^. Over the last decade, substrate culture has expanded rapidly in China^5,6^. Irrigation management of substrate cultures is of great importance because irrigation is the only source of water required by crops and the water-holding capacity of the substrate is very low^3,7,8^. In practice, most irrigation is managed based on the experiences of growers and advisors. Timer-based methods are commonly used in the irrigation management of substrate cultures in solar greenhouses in China. This can easily lead to plants suffering from water stress caused by inadequate irrigation, or roots suffering from hypoxia due to excessive irrigation, adversely affecting plant growth and fruit production^2,3,9^. Noncirculating systems are more commonly used for irrigation in coconut coir cultures because of the difficulty in filtration and disinfection of the discharge liquid^10^. Excessive irrigation also results in the wastage of water and fertilizer and causes environmental pollution, which increases the pressure on water scarcity and environmental protection. Therefore, precise irrigation scheduling for coconut coir cultivation in solar greenhouses is urgently required owing to its potential to promote yield, decrease water used for irrigation, and improve water-use efficiency.

Preparing an irrigation schedule involves determining when (irrigation frequency) and how much to irrigate (irrigation amount)^11,12^. Irrigation frequency is related to the climate and crop development stage. The irrigation amount includes the water required to maintain maximum rates of crop transpiration without water stress and an additional amount to manage substrate salinity and deal with application uniformity^7,12^. However, existing studies on the effects of irrigation frequency on crop production are inconclusive^11^. The measurements of substrates, atmospheric variables, and plants closely related to crop transpiration can directly help make decisions or provide information for calculating the irrigation frequency and amount^13^. In recent years, the most commonly researched approaches for irrigation include 1) the use of soil/substrate moisture sensors^4,14,15^, 2) the estimation of crop water requirements based on climatic data^2,8,16^, and 3) the use of weighing lysimeters^17–19^ or plant sensors^10^.

Soil moisture sensors can be read with continuous automatic data collection to obtain detailed information on the dynamics of water use by crops, which can be used to manage irrigation^9,20,21^. The simplest method is to compare the values of the soil moisture sensor with the selected lower and upper limits of soil moisture to initiate and stop irrigation^9,12^.The appliance of dielectric sensors in substrate has been documented by many research^7,12,22–24^. Capacitive or frequency-domain reflectance (FDR)-based moisture sensors have the advantages of low cost, good reliability, and easy maintenance, and are widely used in research applications and commercial cultivation irrigation management^7,9,25^. Moreover, the approaches for irrigation using climatic data are based on energy balance and bring water supply more closely match to crop transpiration. The accumulated radiation method, which initiates irrigation based on measurements of the integral of solar radiation over the canopy, is a relatively easy approach based on climatic data^2,23^.The method is commonly used in greenhouses in the Netherlands, Italy, and Turkey and in substrate cultivation of glass greenhouse in China^2,7,12,26^.The crop evapotranspiration (ETc) estimated from ETc = ETo $\times$ Kc, where ETo is the reference crop evapotranspiration estimated by the Penman-Monteith method and Kc is the crop coefficient, is important to correctly quantify crop irrigation requirements^27^.Many commercial weather stations provide precise and real-time environmental data on a scale of hours or even minutes. These data are consistent with the requirements for hourly ETo calculation^12,28^.The irrigation scheduling by estimating ETc using the hourly PM-ETo equation, which provides numerous small irrigations each day, is a more scientific method based on climate parameters for substrate culture. However,The response of plant transpiration rate to solar radiation may vary with variations in crop species, developmental stages, and the environment^29–31^.The accumulated radiation threshold for triggering irrigation or irrigation amount should be adjusted for specific application scenarios^2,10,26^.The three constant Kc values of the standard FAO method, each for a fixed developmental stage, are unsuitable for greenhouse-grown crops because of variability in crop training systems and management practices, planting dates, length of the cropping cycle, plant density, and cultivars^7,12,15,32^. Using the three constant Kc values of the standard FAO method to estimate ETc may impact the accuracy of irrigation amount. Therefore, to enhance irrigation management for coconut coir cultivation in solar greenhouses, it is necessary to improve irrigation schedules.

The integration of meteorological parameters and soil moisture sensors allows for the estimation of irrigation levels using energy balance in combination with site-specific adaptive responses to sensors^15,33^.In substrate cultivation, the amount of irrigation and discharge can be easily measured manually or automatically. The ETc can be calculated using the water balance formula and used to divide by ETo to obtain the actual Kc, which can improve the accuracy of crop evapotranspiration estimation for precise irrigation. Therefore, we developed two irrigation schedules. One is a composite irrigation system that combines accumulated radiation and soil moisture sensors (Rn-VWC), and the other is an improved method based on estimating ETc using the hourly PM-ETo equation with an improved calculation of Kc values (T-ETc). Furthermore, the soil moisture sensor-based method (T-VWC)^34^, developed in our previous work, was used as a reference. The analysis included irrigation, evapotranspiration, substrate water content, growth and yield characteristics, and IWP. The goal is to establish a precise irrigation schedule for coconut coir cultivation in solar greenhouses to ensure cucumber yield and improve IWP in China.

## Methods

### Experimental conditions

This research was carried out at the Xiaotangshan National Precision Agriculture Experiment Station in Beijing, China (40$^{\circ }$10’43” N, 116$^{\circ }$26’39” E) from March 15 to June 19, 2017. The experimental greenhouse was 28 m long and 7.5 m wide, with a wall made of brick-concrete and brackets constructed with welded galvanized steel pipes and covered with a transparent plastic film. To avoid excessive temperatures within the structure, the greenhouse was screened with a 50% shading net from 10:00 to 15:00 on sunny days from May 16 to June 19.

**Figure 1:**
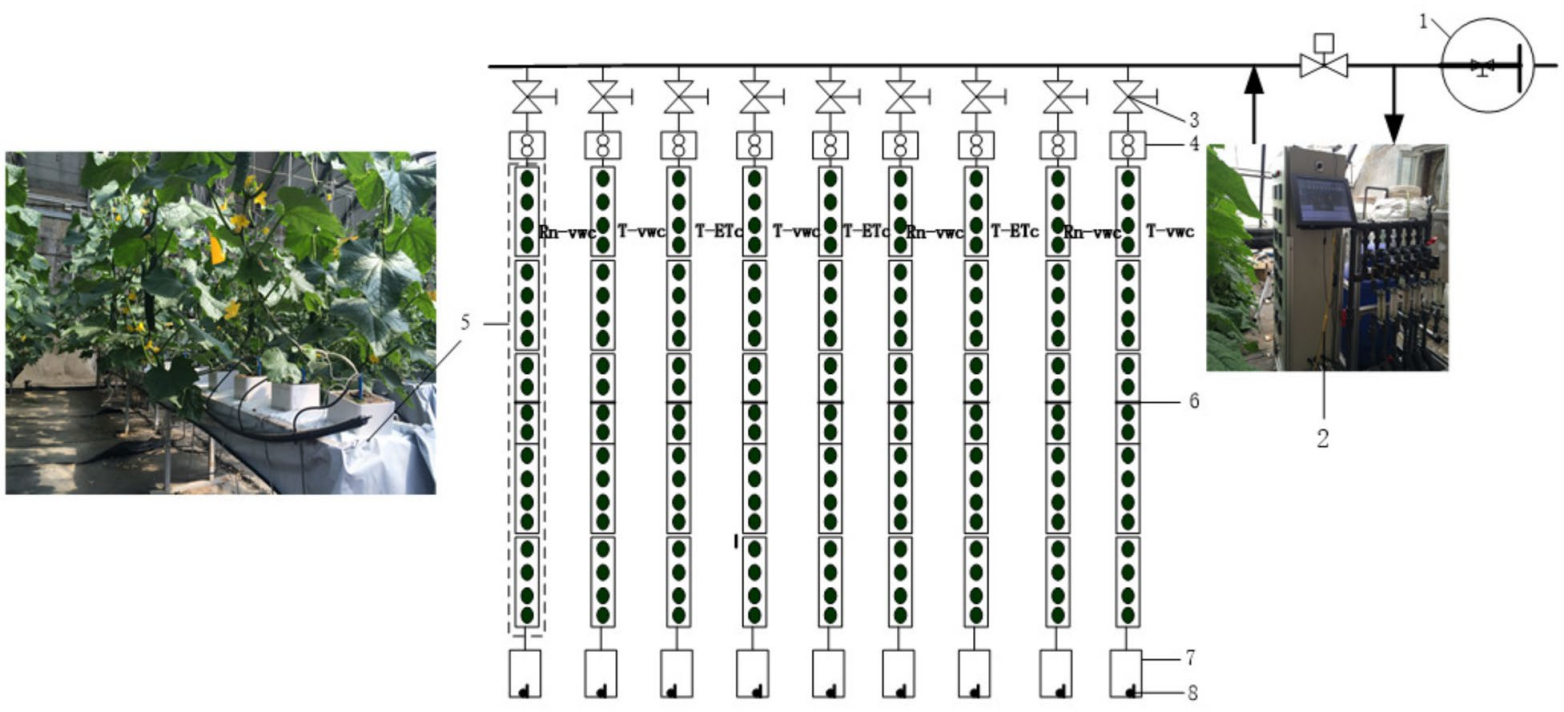
Schematic diagram of the experimental set-up: (1) ressure-regulated water source. (2) Automatic fertigation application. (3) Electric valves. (4) High-precision flow meters. (5) H-type cultivation systems. (6) Frequency-domain reflectance (FDR) sensors. (7) Buckets for collecting discharge. (8) Electrical conductivity and water level sensors. T-VWC: the soil moisture sensor-based method; Rn-VWC: the accumulated radiation combined with soil moisture sensor-based method; T-ETc: the crop evapotranspiration (ETc) estimated method using the hourly PM-ETo equation with an improved calculation of Kc.

The H-type substrate cultivation system was 500 cm long, 24 cm wide, and 40 cm high and was equipped with a cultivation tank with a hole for drainage at the end (Fig. 1(5)). A bucket was connected to the hold via a plastic hose to collect the discharged water. Coconut coir slabs [ 100 cm (length) $\times$ 20 cm (wide) $\times$ 8 cm (height)] were composed of 70% coconut chunks (10–20 mm) and 30% coconut bran (0-6 mm) with EC $< 1.00$ mS cm$^{-1}$ and pH 5.8–6.8. The bulk density, porosity air-water ratio, and organic matter of the coconut coir slab were 0.14 g cm$^{-3}$, 87.89%, 0.26, and 86.4%, respectively. Coconut coir slabs were wrapped with a polyethylene (PE) film on six sides to avoid the evaporation of substrate water. Irrigation was controlled using an automatic fertigation applicator developed by the Intelligent Equipment Research Center of the Beijing Academy of Agriculture and Forestry Sciences^34^. Yamazaki cucumber nutrient solution formula was used in this study and was applied using a drip irrigation system with in-line emitters of 2 L h$^{-1}$ discharge. The emitter distance was 25 cm and was associated with a single row of plants.

The cucumber variety used in the experiment was “Zhongnong 26”, and cucumbers were transplanted on March 15 and harvested from April 19 to June 19. Cucumbers were arranged in two rows for each cultivation system, and 10 plants were planted in each row at a planting density of 3 plants m$^{-2}$.

### Experimental design

Two irrigation schedules were developed: a complex irrigation schedule combining accumulated radiation and soil moisture sensors (Rn-VWC) and another irrigation schedule based on estimating ETc by the hourly PM-ETo equation with an improved calculation of Kc values (T-ETc). The soil moisture sensor-based method (T-VWC)^34^, which was developed in our previous research and significantly reduces irrigation amount and improves IWP compared with Timer-based scheduling, was used as a reference. In the T-VWC treatment, irrigation frequency and amount were determined hourly from 7:00 to 18:00. Irrigation was triggered when the value recorded by the soil moisture sensor was lower than the maximum substrate water content ($VWC_{max}$, m$^{3}$ m$^{-3}$). The amount of irrigation was determined by the substrate water content read by the sensor (*VWC*, m$^{3}$ m$^{-3}$).In Rn-VWC treatment, every irrigation event was initiated when the accumulative radiation reached 0.8 MJ m$^{-2}$, and the calculation of irrigation amount was consistent with T-VWC. The irrigation amount of the T-ETc treatment was dependent on the hourly ETc ($ETc_{h}$) calculated by multiplying Kc by the hourly ETo ($ETo_{h}$), where $ETo_{h}$ was estimated using the standardized reference evapotranspiration equation (ASCE-PM)^28^, and Kc was obtained from the daily actual evapotranspiration ($ET_{d}$) and daily ETo ($ETo_{d}$) of the previous day. Irrigation was triggered when $ETc_{h}$ reached or exceeded 0.4 mm to avoid a small amount for a single irrigation; otherwise, irrigation was not performed temporarily, and this $ETc_{h}$ was accumulated into the next time. Furthermore, the coefficient *k* was incorporated to aid in determining the irrigation amount to ensure proper drainage for managing substrate salinity. The value of *k* was determined based on the variance between the electrical conductivity (EC) of irrigation and drainage.^2,35,36^. Descriptions of the three irrigation schedules were presented in Table 1.

$M_1$ in Table 1 was the calculated irrigation amount used for the actual irrigation amount (*Ir*) of the T-VWC and Rn-VWC treatments,and could be described as follows:

1 $$\begin{aligned} M_1 = 0.001 (VWC_{max} - VWC) V p/\eta \end{aligned}$$

where $VWC_{max}$ is 0.507 (m$^{3}$ m$^{-3}$), *V* is the volume of the substrate to be irrigated (m$^{3}$), *p* is the wetting ratio of the substrate, $\eta$ is the irrigation efficiency.In substrate cultivation, the small volume of the substrate is distributed by the root system of the crop, requiring complete wetting through irrigation. Therefore, *p* in this study was 100%. The nutrient solution was delivered to the substrate without any loss, $\eta$ was set at 1.0.

*s* in Table 1 was the area of the cultivation plot (m$^{2}$). The $ETc_{h}$ was determined using Eq. (2).

2 $$\begin{aligned} ETc_{h}=K_c ETo_{h} \end{aligned}$$

where $K_c$ can be obtained with an improved calculation as follows:

3 $$\begin{aligned} K_c=ET_{d}/ETo_{d} \end{aligned}$$

The *ET* was the actual evapotranspiration calculated by the water balance method^37^. The change in substrate water content was too small to be neglected compared to the evapotranspiration and runoff did not occur. Therefore, The *ET* and $ET_{d}$ in Ep.3 were determined using Eqs. (4) and (5),respectively.

4 $$\begin{aligned} ET=(Ir-D)/s \end{aligned}$$

where *s* is the area of the cultivation plot (m$^{2}$), *Ir* is the amount of irrigation (L), and *D* is the amount of discharge (L).

5 $$\begin{aligned} ET_{d}=(Ir_{d}-D_d)/s \end{aligned}$$

where $Ir_d$ is the daily amount of irrigation (L), and $D_d$ is the daily amount of discharge (L).

Because coconut coir slabs were wrapped with PE film on six sides to avoid the evaporation of substrate water, it was considered that the actual evapotranspiration (ET) was equal to the transpiration of cucumber plants in this study. The three irrigation schedules were programmed and imported into the operating system of the automatic fertigation system and started at the first cucumber fruit site (April 14), and The Timer-based method (0.02 L plant$^{-1}$ h$^{-1}$) was applied before.

The treatments were arranged in a completely randomized block design with three replicates per treatment. One H-type cultivation system was a replicate equipped with a branch pipe connected to the main pipe through an electric valve and a high-precision flowmeter, which was used to ensure independent irrigation of each cultivation system. At the center of each cultivation system, an EC-5 sensor (METER Group, Inc., USA) was installed between the two plants in the coconut coir slabs to measure the substrate water content. The EC of the irrigation and discharge nutrient solutions were measured using electrical conductivity (EC) sensors. The amounts of irrigation and discharge were measured using high-precision flow meters and water lever sensors installed in the discharge collection bucket, respectively. Irrigation frequency was obtained by recording the opening and closing of the electric valves. All the electric valves, high-precision flow meters, and sensors were connected to an automatic fertigation system. The schematic diagram of the experimental set-up is shown in Fig. 1. The VWC, EC, and water level data from the sensors were collected every 5 s.

### Sampling and measurements

The solar radiation, temperature, and relative humidity were measured every 5 s using a net radiation sensor (TBQ-2) and an air temperature and humidity sensor (PTS-3) developed by Jinzhou Sunshine Meteorological Science Co., Ltd., China.

**Table 1:** Treatments for the three irrigation schedules.

Irrigation scheduling	Irrigation frequency		Irrigation amount	
T-VWC Rn-VWC T-ETc	07:00–18:00 on the hour $\sum {Rn_i \le 0.8~ \mathrm{MJ~ m}^{-2}}$ 07:00-18:00 on the hour	$VWC \le VWC_{max}$ $VWC \le VWC_{max}$ $ETc_{h} \ge 0.4~ \textrm{mm}$	$Ir=k \cdot M_1$ $Ir=k \cdot M_1$ $Ir=k \cdot s \cdot ETc_{h}$	If $EC_{\textrm{D}} - EC_{\textrm{Ir}} \le$ $1.00~ {\textrm{mS~ cm}}^{-1}$^35^ $,~ k = 1.0; ~ \textrm{else} ~k = 1.3$^2,35^

The leaf was marked at the beginning of its expansion, and its area was calculated by measuring the length and width^38^. Stem diameter 1 cm below the marked leaf was measured with a vernier caliper. The fruits were harvested when they reached normal size. The yield and number of cucumbers from each cultivation system were determined at the beginning (April 19 to April 30), middle (May 1 to June 5) and end (June 6 to June 19) of the harvest, and the mean fruit weight and total yield were calculated. The soluble protein, soluble sugar, vitamin C, and NO$_3^{-}$-N contents of five fruits per replicate were measured at mid-harvest to compare fruit quality^39^. Drainage rate (DR) and IWP (fresh weight/irrigation amount) were also calculated with Eqs. (6) and (7),(respectively.

6 $$\begin{aligned} DR= & {} D/Ir \end{aligned}$$

7 $$\begin{aligned} IWP=Y/I \end{aligned}$$

where *Y* is total yield (t ha$^{-1}$), and *I* is the total amount of irrigation (m$^{3}$ ha$^{-1}$).

### Statistical analysis

Statistical analyses were conducted by one-way analysis of variance (ANOVA), using SPSS 17.0 software (SPSS Inc., Chicago, USA).The differences among mean values were established by Duncan’s multiple range test at P $< 0.05$.

## Results and analysis

### Irrigation, evapotranspiration, and drainage at harvest of cucumber

The dates related to the daily irrigation and evapotranspiration, and drainage of the three irrigation schedules during harvest were listed in Table 2.The daily number of irrigation events varied according to irrigation scheduling, and the highest and lowest were recorded at 14 and 5 times at the beginning and end of harvest, respectively, for the Rn-VWC treatment.The daily number of irrigation under the T-VWC and T-ETc treatments were 12 times and 9-10 times. The daily amount of irrigation ($Ir_{d}$) was recorded as 1.70–2.00 L plant$^{-1}$, 0.85–2.03 L plant$^{-1}$, and 1.12–1.41 L plant$^{-1}$ in the T-VWC, Rn-VWC, and T-ETc treatments, respectively. Similar to the irrigation events, the highest and lowest $Ir_{d}$ were recorded at the beginning and end of the harvest, respectively, in the Rn-VWC treatment, and the difference between the three stages of the harvest was small in the T-VWC and T-ETc treatments. At the beginning and middle of harvest, $Ir_{d}$ in the T-ETc treatment was lower than that in the other treatments.

**Table 2:** The effect of irrigation scheduling on the daily number and amount of irrigation together with evapotranspiration and drainage rate at the beginning, middle and end of harvest.

		T-VWC	Rn-VWC	T-ETc
The beginning of harvest April 19 to April 30	Number (times)	12	14	9
	$Ir_{d}$ (L plant$^{-1}$)	1.96	2.03	1.29
	$ET_{d}$ (mm)	4.63	4.66	2.94
	$D_{d}$ (L plant$^{-1}$)	0.34	0.40	0.26
The middle of harvest May 1 to June 5	Number (times)	12	9	9
	$Ir_{d}$ (L plant$^{-1}$)	2.00	1.40	1.12
	$ET_{d}$ (mm)	3.79	3.03	2.17
	$D_{d}$ (L plant$^{-1}$)	0.67	0.34	0.36
The end of harvest June 6 to June 19	Number (times)	12	5	10
	$Ir_{d}$ (L plant$^{-1}$)	1.70	0.85	1.41
	$ET_{d}$ (mm)	3.63	1.91	2.58
	$D_{d}$ (L plant$^{-1}$)	0.43	0.18	0.50

T-VWC: the soil moisture sensor-based method; Rn-VWC: the accumulated radiation combined with soil moisture sensor-based method; T-ETc: the crop evapotranspiration (ETc) estimated method using the hourly PM-ETo equation with an improved calculation of Kc. Number: daily number of irrigation; $Ir_{d}$: daily amount of irrigation; $ET_{d}$: daily actual evapotranspiration; *D*_d_: daily drainage.

Because irrigation is the only source of water for cucumber plants, the daily evapotranspiration ($ET_{d}$) were very similar to the irrigation. The maximum and minimum $ET_{d}$ were 4.66 and 1.91 mm, at the beginning and end of the harvest in Rn-VWC treatment, respectively. Moreover, the T-ETc treatment had the lowest $ET_{d}$ at the beginning and middle of harvest, with figures for 2.17 and 2.94 mm, respectively, and at the end of harvest was lower than that in the T-VWC treatment.

The daily drainage ($D_d$) under the T-VWC, Rn-VWC, and T-ETc treatments during the experiment ranged from 0.34-0.43 L plant$^{-1}$),0.18-0.40 L plant$^{-1}$),0.26-0.50 L plant$^{-1}$), respectively.Thus,the drainage rate (DR) was within an appropriate range and was calculated as 17.49–33.48%, 19.57–24.47%, and 19.95–35.83%, respectively. The differences in DR among the different treatments at the beginning and end of harvest were the smallest and the largest, respectively.

### Irrigation, evapotranspiration, and substrate water content under different weather conditions

#### The solar radiation, air temperature, and relative humidity Of different weather conditions

**Figure 2:**
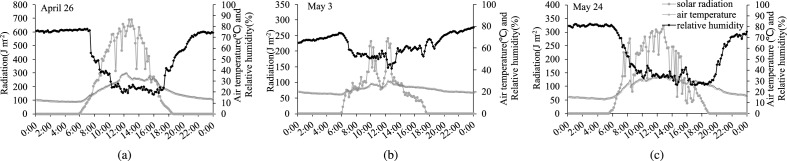
Diurnal curves of indoor solar radiation, air temperature, and relative humidity. (**a**) Indoor solar radiation, air temperature, and relative humidity on April 26 (sunny); (**b**) Indoor solar radiation, air temperature, and relative humidity on May 3 (cloudy); (**c**) Indoor solar radiation, air temperature, and relative humidity on May 24 (sunny and shading screens used).

The solar radiation, air temperature, and relative humidity inside the greenhouse on April 26 (sunny), May 3 (cloudy), and May 24 (sunny and shading screens used) are plotted in Fig. 2. The daily solar radiation in these three days was 15.45 MJ m$^{-2}$, 3.98 MJ m$^{-2}$, and 7.56 MJ m$^{-2}$, respectively, and the mean air temperature and relative humidity were 20.38 $^{\circ }$ C, 22.08 $^{\circ }$ C, and 23.40 $^{\circ }$ C and 52.51%, 46.10%, and 54.16%, respectively. The maximum values of solar radiation and air temperature on April 26 and May 24 reached 691 J m$^{-2}$ s$^{-1}$ and 37.12 $^{\circ }$ C and 322 J m$^{-2}$ s$^{-1}$ and 34.02 $^{\circ }$ C, respectively, indicating that the use of shading screens on May 24 effectively reduced the solar radiation inside the green-house and kept the maximum air temperature within 35 $^{\circ }$ C.

**a**) Indoor solar radiation, air temperature, and relative humidity on April 26 (sunny); (**b**) Indoor solar radiation, air temperature, and relative humidity on May 3 (cloudy); (**c**) Indoor solar radiation, air temperature, and relative humidity on May 24 (sunny and shading screens used).

#### Irrigation and evapotranspiration under different weather conditions

**Figure 3:**
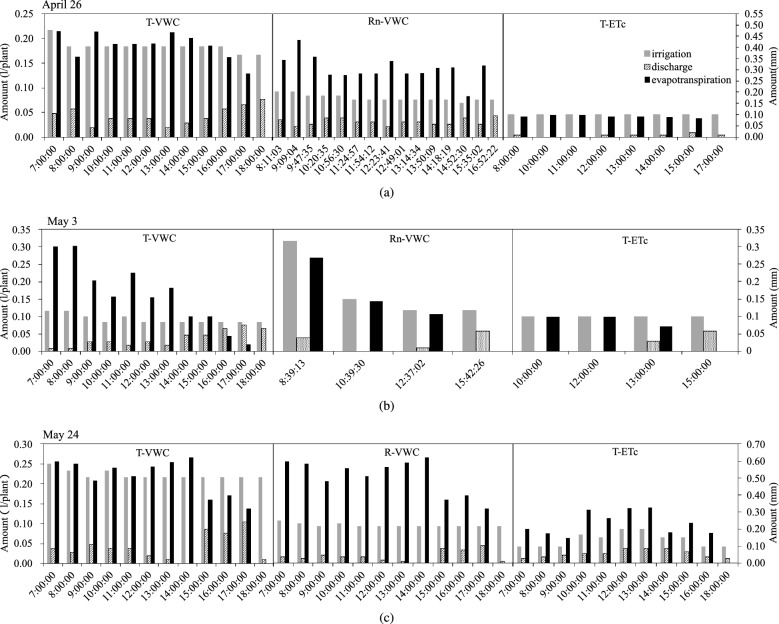
Irrigation and discharge amount together with evapotranspiration under different irrigation schedules. T-VWC: the soil moisture sensor-based method; Rn-VWC: the accumulated radiation combined with soil moisture sensor-based method; T-ETc: the crop evapotranspiration (ETc) estimated method using the hourly PM-ETo equation with an improved calculation of Kc. (**a**) Irrigation and discharge amount together with evapotranspiration on April 26 (sunny); (**b**) irrigation and discharge amount together with evapotranspiration on May 3 (cloudy); (**c**) irrigation and discharge amount together with evapotranspiration on May 24 (sunny and shading screens used).

The irrigation frequency of T-VWC and T-ETc treatments was determined using Timer (7:00–18:00) and had the auxiliary condition of “ETc $\ge$ 0.4 mm for starting irrigation” under the T-ETc treatment. Figure 3 shown that the irrigation events in the T-ETc treatment were 8, 4, and 11 times, which were less than that in the T-VWC treatment (12 times). For the Rn-VWC treatment based on accumulated radiation to determine the irrigation frequency, the irrigation period was shortened with an increase in solar radiation. On April 26 (sunny), the irrigation frequency from 10:00 to 15:00 was significantly higher than that during other periods, and had the maximum number of irrigation events with 15 times; the minimum number of irrigation events was four times, recorded on May 3 (cloudy).

Owing to the amount of irrigation (Ir) based on the soil moisture sensor, the T-VWC and Rn-VWC treatments had the largest amount for the first daily irrigation event (Fig. 3). This might be because the root system absorbed water from the substrate for plant transpiration and metabolism, and no irrigation occurred at night, which led to a decrease in substrate water content. The fluctuations in the amounts between other irrigation events were small. The Ir of the T-ETc treatment was 0.10 L plant$^{-1}$ on April 26 and May 3, and showed a single peak curve similar to radiation and temperature on May 24, with a maximum value of 0.20 L plant$^{-1}$.

The ET values for the three irrigation schedules were shown in Fig. 3. Daily variations in ET were analyzed. The ET of the T-VWC and Rn-VWC treatments were higher in the first irrigation cycle, which might be related to a higher amount of irrigation. The ET in the T-VWC treatment gradually decreased in the afternoon, which differed from the variation in irrigation amount. However, the daily variation of ET was consistent with that of irrigation amount in the T-ETc treatment. Additionally, both the ET of the T-VWC treatment on May 3 (cloudy) and the ET of the T-ETc treatment on May 24 (sunny with shading screens used) were significantly lower than their irrigation amount, resulting in a high DR (40.75% and 40.88%, respectively). The Rn-VWC treatment had a similar effect on two sunny days. This suggests that the T-VWC treatment on cloudy day, the Rn-VWC treatment on sunny day, and the T-ETc treatment on sunny day with shading screens used were over-irrigated.

#### Substrate water content under different weather conditions

**Figure 4:**
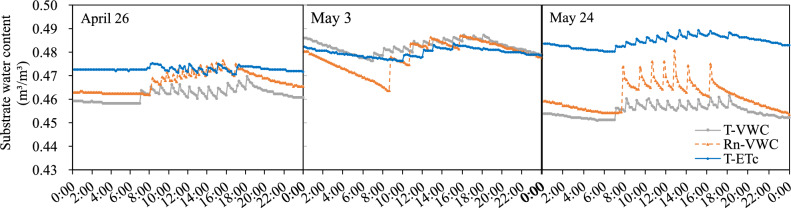
The substrate water content diurnal curves under different irrigation schedules. T-VWC: the soil moisture sensor-based method; Rn-VWC: the accumulated radiation combined with soil moisture sensor-based method; T-ETc: the crop evapotranspiration (ETc) estimated method using the hourly PM-ETo equation with an improved calculation of Kc. (left) The substrate water content diurnal curves on April 26 (sunny); (middle) The substrate water content diurnal curves on May 3 (cloudy); (right) The substrate water content diurnal curves on May 24 (sunny and shading screens used).

The changes in substrate water content under different irrigation schedules were shown in Fig. 4. The substrate water content in the T-VWC and Rn-VWC treatments were always lower than the maximum substrate water content during the experiment. Because the low indoor light radiation on May 3 and 24 increased the irrigation interval, and the strong indoor light radiation on April 26 led to frequent irrigation, the substrate water content of the Rn-VWC treatment had the largest fluctuation. The substrate water content of the T-ETc treatment was relatively stable, which might be because the irrigation supply tended to synchronize with evapotranspiration. In addition, there was a significant increase in the substrate moisture content of the T-VWC and Rn-VWC treatments on April 26 and the T-ETc treatment on May 24, which might be due to excessive irrigation.

### Effect of irrigation scheduling on plant growth of cucumber

**Figure 5:**
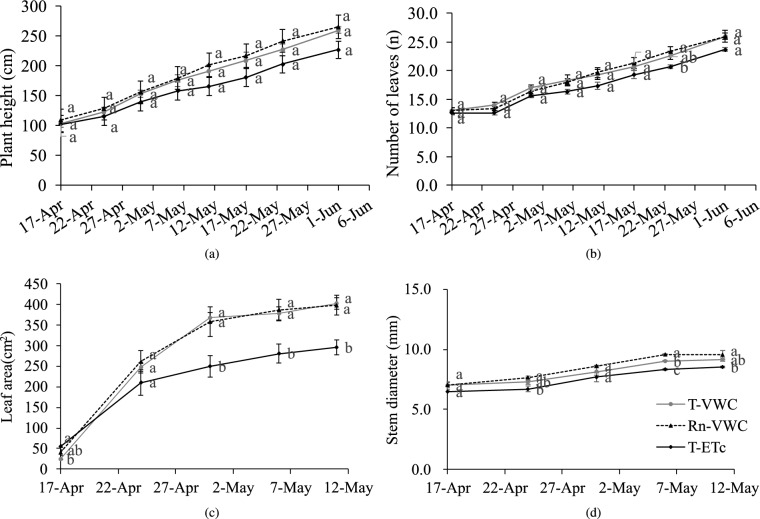
Dynamics of the cucumber plant growth under different irrigation schedules. T-VWC: the soil moisture sensor-based method; Rn-VWC: the accumulated radiation combined with soil moisture sensor-based method; T-ETc: the crop evapotranspiration (ETc) estimated method using the hourly PM-ETo equation with an improved calculation of Kc. (**a**) Plant height; (**b**) number of leaves; (**c**) leaf area; (**d**) stem diameter. Vertical bars represent the standard error of the mean (n = 3). Different letters after standard error of mean indicate a significant difference between treatments (Duncan’s test, P < 0.05).

As shown in Fig. 5, the plant height and the number of leaves increased continuously from April 17 to June 1. The area of the marked leaves and stem diameter below the marked leaf increased rapidly at the beginning of the measurement period, after April 31 it increased slowly. The height, number of leaves, leaf area, and stem diameter of the T-VWC and Rn-VWC treatments were higher than those of the T-ETc treatment, and the differences of the height and the number of leaves after April 31 and the leaf area after April 25 were more obvious. But there was no difference between the T-VWC and Rn-VWC treatments.

### Yield and quality of cucumber

Table 3 listed the cucumber number, mean weight, and yield of the T-VWC, Rn-VWC, and T-ETc treatments at each harvesting stage. There were not significantly different except that the mean weight of fruit in the T-ETc treatment at the last fruit stage was significantly higher than that in the Rn-VWC treatment (P $< 0.05$). Because the number, mean weight, and yield in the Rn-VWC treatment were the lowest at all harvest stages, its total yield (58.98 t ha$^{-1}$) was lower than that of the T-VWC (66.77 t ha$^{-1}$) and T-ETc (64.54 t ha$^{-1}$) treatments (Table 3). The T-VWC treatment had the highest total yield but the difference between the three treatments did not reach a significant level (P $< 0.05$). Table 4 shown that the vitamin C content of fruits in the T-VWC treatment was significantly higher than that in the Rn-VWC treatment, whereas the contents of soluble protein, soluble sugar, and NO$_3^{-}$-N were not significantly different.

**Table 3:** The effect of irrigation scheduling on the number and mean weight of fruits and the yield of cucumber at the beginning, middle, and end of harvest.

		T-VWC	Rn-VWC	T-ETc
The beginning of harvest April 19–April 30	Number (plant$^{-1}$)	1.73 ± 0.93a	1.67 ± 0.18a	1.70 ± 0.05a
	Mean weight (g)	148.67 ± 4.91a	142.00 ± 2.52a	153.33 ± 7.45a
	Yield (t ha$^{-1}$)	7.75 ± 0.63a	7.10 ± 0.81a	7.80 ± 0.19a
The middle of harvest May 1–June 5	Number (plant$^{-1}$)	8.35 ± 0.43a	7.83 ± 0.37a	7.83 ± 0.22a
	Mean weight (g)	197.67 ± 3.76a	189.33 ± 4.33a	196.67 ± 2.91a
	Yield (t ha$^{-1}$)	49.59 ± 3.33a	44.56 ± 2.96a	46.26 ± 1.79a
The end of harvest June 6–June 19	Number (plant$^{-1}$)	1.52 ± 0.12a	1.28 ± 0.12a	1.45 ± 0.10a
	Mean weight (g)	207.67 ± 6.23ab	187.67 ± 6.84b	222.00 ± 6.43a
	Yield (t ha$^{-1}$)	9.43 ± 0.62a	7.28 ± 0.91a	9.68 ± 0.87a
	Total yield (t ha$^{-1}$)	66.77 ± 4.36a	58.98 ± 4.35a	64.54 ± 3.22a

T-VWC: the soil moisture sensor-based method; Rn-VWC: the accumulated radiation combined with soil moisture sensor-based method; T-ETc: the crop evapotranspiration (ETc) estimated method using the hourly PM-ETo equation with an improved calculation of Kc. Values are given as means ± standard error of means (n = 3). Different letters after standard error of mean indicate a significant difference between treatments (Duncan’s test, P $<0.05$).

**Table 4:** The effect of irrigation scheduling on the cucumber fruit quality.

Treatments	Soluble protein (%)	Soluble sugar (%)	Vitamin C (mg kg$^{-1}$)	NO$_3^{-}$-N (mg kg$^{-1}$)
T-VWC	0.88 ± 0.06a	2.93 ± 0.16a	160.00 ± 10.00a	91.77 ± 4.89a
Rn-VWC	0.88 ± 0.02a	2.62 ± 0.07a	104.27 ± 13.92b	90.23 ± 2.46a
T-ETc	0.79 ± 0.06a	2.92 ± 0.05a	126.67 ± 19.34ab	91.80 ± 3.43a

T-VWC: the soil moisture sensor-based method; Rn-VWC: the accumulated radiation combined with soil moisture sensor-based method; T-ETc: the crop evapotranspiration (ETc) estimated method using the hourly PM-ETo equation with an improved calculation of Kc. Values are given as means ± standard error of means (n = 3). Different letters after standard error of mean indicate a significant difference between treatments (Duncan’s test, P $<0.05$).

### Total irrigation amount and irrigation water productivity

**Figure 6:**
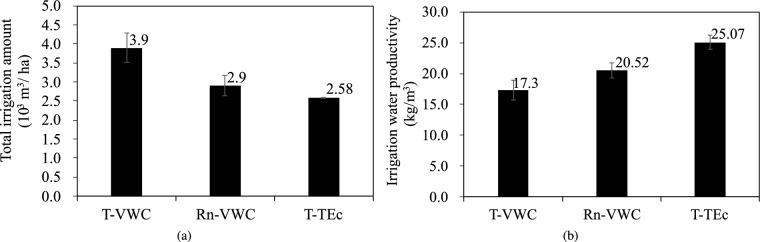
Total irrigation amount and irrigation water productivity under different irrigation schedules. T-VWC: the soil moisture sensor-based method; Rn-VWC: the accumulated radiation combined with soil moisture sensor-based method; T-ETc: the crop evapotranspiration (ETc) estimated method using the hourly PM-ETo equation with an improved calculation of Kc. (**a**) Total irrigation amount; (**b**) irrigation water productivity. Vertical bars represent the standard error of the mean (n = 3).

The Total irrigation amount (I) determined for the treatments with different irrigation schedules were summarized in Fig. 6 (a). The I under the T-VWC, Rn-VWC, and T-ETc treatments were 3,901.92 m$^{3}$ ha$^{-1}$, 2897.15 m$^{3}$ ha$^{-1}$, and 2573.88 m$^{3}$ ha$^{-1}$, respectively. The T-VWC and T-ETc treatment had the highest and the lowest values. Compared with the T-VWC treatment, *I* for the Rn-VWC and T-ETc treatments significantly decreased by 25.75% and 34.04%, respectively, and there was significant difference between the T-VWC and T-ETc treatments (P $<0.05$).

As shown in Fig. 6b, the irrigation water productivity (IWP) under the T-VWC, Rn-VWC, and T-ETc treatments were 17.37 kg m^−3^, 20.52 kg m^−3^, 25.07 kg m^−3^, respectively. Compared with the T-VWC treatment, the IWP under the Rn-VWC and T-ETc treatments increased by 18.13% and 44.33%, respectively. The T-ETc treatment had the highest IWP. Moreover, there was a significant difference between T-ETc treatment and T-VWC treatment (P $< 0.05$). This was linked to the calculation of irrigation amount based on the estimation of ETc in the T-ETc treatment, which resulted in no loss of yield, but a significant decrease in irrigation and evapotranspiration.

## Discussion

### Effect of irrigation scheduling on irrigation

Scientific irrigation scheduling is needed to synchronize the supply with the real water requirements of substrate-cultivated crops and to maintain an appropriate substrate water content and DR to protect the crop from water and salt stress. The DR was generally 15–30% and it could increase to 30–35% if necessary^2,36^. In addition, owing to the limited volume and low water retention of the substrate, irrigation needed to be small and frequent to ensure good plant growth. The results of this study shown that the daily irrigation frequency under the T-VWC and T-ETc treatments was 12 times and 9–10 times, the Ir was 1.70–2.00 L plant$^{-1}$ and 1.12–1.41 L plant$^{-1}$, and the DR was 17.49–33.48% and 19.95–35.83%, respectively. The irrigation frequency under the Rn-VWC treatment decreased with the weakening of solar radiation while increased with the strengthening of solar radiation in the greenhouse (Fig. 3). This led to a high amount of irrigation at the beginning of the harvest period (Table 2), a decrease in irrigation at the middle and end of the harvest period (Table 2) and a large fluctuation in the water content of the substrate (Fig. 4), which is consistent with the results of Wei et al^26^. Compared with the Rn-VWC treatment, the T-VWC and T-ETc treatments provided more suitable irrigation management for cucumbers in coconut coir culture. As the coconut coir slab is a coarse substrate with high porosity, finger flow is easily formed under drip irrigation, which reduces the water content of the substrate at a distance from the emitter or the time lag to reach the same water content^4,24^.Moreover, the soil moisture sensor represents a “point measurement” in space^4,11,20^. The placement of soil moisture sensor directly affects the efficiency of irrigation scheduling^4^. the T-VWC treatment had a higher daily irrigation amount (Table 2) and fluctuations in substrate water content (Fig. 4) than the T-ETc treatment. These might be related to the fact that the irrigation amount of the T-VWC treatment was based on soil moisture sensors, which were arranged in the middle of the adjacent emitters. The T-ETc treatment made the change in of irrigation amount tend to the actual transpiration of cucumber plants (Fig. 3) and then reduced the total irrigation amount by 34.04% compared with the T-VWC treatment (Table 2). Therefore, the T-ETc treatment was better than the T-VWC treatment in this study. The T-VWC treatment based on soil moisture sensor needs to further optimizing the installation position of the sensor to improve irrigation efficiency.

### Effect of irrigation scheduling on evapotranspiration

In general, crop transpiration increases with irrigation^2,11,33^. In this study, evapotranspiration was equal to the transpiration of the cucumber plants because the six sides of the coconut coir slabs were wrapped with PE films to avoid the evaporation of substrate water. The difference in $ET_{d}$ among the different treatments at each harvest stage was consistent with that in Ir. The $ET_{d}$ of the T-ETc treatment was the lowest at the beginning and middle of the harvest and was inferior to that of the T-VWC treatment at the end of the harvest (Table 2). In addition, the T-ETc treatment with low irrigation had the smallest number of leaves and leaf area, which further reduced the transpiration from cucumber plants. It is often assumed that the water requirements of greenhouse-grown crops are equivalent to those of crop evapotranspiration. This indicates that the daily variation tendency of irrigation amount should be consistent with evapotranspiration.^12^. In the three meteorological environments of this experiment, the ET of the T-VWC treatment decreased with the weakening of solar radiation in the afternoon, especially on cloudy days, which was different from the irrigation amount, with a very small variation. The daily variation of ET in the Rn-VWC and T-ETc treatments had the same tendency as that of the irrigation amount (Fig. 3). Shin et al^31,33^ found that the transpiration rate was not always proportional to the light intensity and was almost constant when the light intensity exceeded 200 J s$^{-1}$ m$^{-1}$, suggested that the cumulative radiation threshold used to trigger irrigation should be adjusted appropriately under bright light conditions to avoid over-irrigation. Similar results were observed for the Rn-VWC treatment. During most of the time from 7:00 to 18:00 on April 26 and May 24, the solar radiation in the greenhouse was above 200 J s$^{-1}$ m$^{-1}$ and the cumulative radiation threshold in Rn-VWC treatment was fixed, which could resulted in excessive irrigation and high DR (over 35%). In addition, the application of environmental control systems in greenhouses affects the correlation between meteorological parameters and crop transpiration^23,31,40^. After using the shading screens, the incident radiation and vapor pressure deficit, the response of leaf transpiration to incident radiation, and the transpiration rate per unit leaf area decreased significantly^29,41^. The ET of cucumber on May 24 in the T-ETc treatment was significantly lower than the irrigation amount (Fig. 3), which may be related to the high $ETc_{h}$ estimated by Eq. (2) due to the use of shading system. Therefore, it is necessary to further investigate the response mechanisms of crop photosynthesis, transpiration, and other physiological activities under the application of greenhouse environmental control systems to provide a theoretical basis for continuing to optimize irrigation methods. The purpose of this study is to obtain a more precise irrigation regime suitable for coconut coir cultivation of cucumber in solar greenhouses. However, the data used for the analysis of irrigation regimes under different meteorological conditions in this study is limited, which needs to be improved in the future studies.

### Effect of irrigation scheduling on the yield of cucumber and IWP

The effect of irrigation scheduling on crop yield in substrate culture depends on irrigation frequency and amount. A higher frequency of irrigation, which could maintain the stability of the substrate water content, helped improve yield^2,9,11,42^. Suyum et al.^9^ found that the fluctuation in substrate water content increases with a decrease in the threshold of substrate water content for irrigation control, resulting in a decline in sweet basil yield. Similar results were observed for the Rn-VWC treatment, with the highest fluctuation in substrate water content and the lowest yield. Especially at the end of harvest, the yield decreased by 22.35–24.81%. The primary response of plants to water stress is the inhibition of cell expansion^43^, which, in turn, affects fruit size and weight. At the end of harvest, the mean weight of fruit in the Rn-VWC treatment was significantly reduced, leading to the lowest yield (Table 3). It is suggested that the large fluctuation in substrate water content may cause the plants to suffer from water stress. However, this result was inconsistent with previous studies on tomatoes^3,11^ and sweet peppers^44^, which showed that a reduction in fruit number was the cause of lower yield under water stress.

According to the characteristics of the crop water production function, the yield increases with an increase in the irrigation amount when water is the limiting factor; if the increase in the irrigation amount is greater than that in crop production, the input of water should be limited^45^ . Simsek et al.^46^ constructed a polynomial equation using cucumber yield and irrigation data from two years and pointed out that excessive irrigation reduces yield. Meric et al.^2^ found that the increase in yield caused by high-frequency irrigation was slower than the increase in the irrigation amount. Hao et al.^11^ reported that the yield of tomatoes increased slowly to approach their plateau when the irrigation amount reached 70% and 90% of cumulative evaporation. The total irrigation amount in the T-VWC treatment was significantly higher than that in the T-ETc treatment, while the total yield was almost the same. This indicated that the greater amount of irrigation in the T-VWC treatment did not contribute to the yield. This finding was consistent with those of above-mentioned studies. Moreover, the plant height, stem diameter, leaf number, and leaf area under the T-VWC treatment were higher than those under the T-ETc treatment. It was suggested that the cucumbers under the T-VWC treatment were biased towards nutrient growth. Due to the pursuit of cucumber yield, T-VWC treatment was considered to provide excessive irrigation, leading to waste of water and fertilizer resources.

IWP is the ratio of yield to irrigation amount and is an important criterion for evaluating production systems^41,47^. In general, IWP increased with the decrease of irrigation amount and the increase of yield. Recep et al.^47^ found that the treatment with the least amount of irrigation resulted in the highest IWP of cucumbers grown in a solar green-house during the springsummer season. In the present study, the decrease in the yield under the Rn-VWC treatment was less than the reduction in the irrigation amount, however, the yield under the T-ETc treatment did not decrease with the reduction in the irrigation amount. Thus, the IWP under the T-ETc treatment was the highest, followed by that under the Rn-VWC treatment.

## Conclusion

In this study, three irrigation schedules for cucumber coconut coir cultures were evaluated. The results shown that the daily irrigation frequency, irrigation amount, and discharge rate under the T-ETc treatment were 9–10 times, 1.12–1.41 L plant$^{-1}$, and 19.95–35.83%, respectively, and the irrigation amount was consistent with the change in ET. Compared to the other two treatments, the total irrigation amount in the T-ETc treatment was the lowest, the cucumber yield was not affected, and IWP was the highest. The T-ETc treatment synchronized irrigation with the transpiration of cucumber plants and saved a substantially larger amount of water and fertilizer. Thus, the method based on estimating ETc using the hourly PM–ETo equation with an improved calculation of Kc values (T-ETc) was a more precise irrigation management method for cucumbers in coconut coir culture.

## Data Availability

The data used to support the findings of this study are included within the article. The study complies with local and national regulations. No collection of seeds or plants are involved in this study. The study complies with local and national regulations. No collection of seeds or plants are involved in this study.
